# Children with Obesity and Asthma: Which Are the Best Options for Their Management?

**DOI:** 10.3390/nu10111634

**Published:** 2018-11-02

**Authors:** Lorenza Di Genova, Laura Penta, Anna Biscarini, Giuseppe Di Cara, Susanna Esposito

**Affiliations:** Pediatric Clinic, Department of Medical and Surgical Sciences, Università degli Studi di Perugia, Piazza Lucio Severi 1, 06132 Perugia, Italy; lory.digenova@gmail.com (L.D.G.); laura.penta@ospedale.perugia.it (L.P.); annabiscarini@libero.it (A.B.); giuseppe.dicara@unipg.it (G.D.C.)

**Keywords:** asthma, obesity, overweight, vitamin D, weight loss

## Abstract

Obesity and asthma are complex disorders related to gene-environment interactions and various lifestyle factors. At present, they represent two of the most significant paediatric health problems worldwide, particularly in industrialized nations. The aim of this narrative review is to evaluate possible therapeutic strategies to manage asthma in children with overweight/obesity. PubMed was used to search for all of the studies published from January 2008 to June 2018 using the following key words: “asthma” and “overweight” or “obesity” or “obese” and “children” or “paediatric”. The literature review showed that growing evidence underlines the existence of an “obese asthma” phenotype characterised by difficult-to-control asthma with additional symptoms, worse control, more frequent and severe exacerbations, reduced response to inhaled corticosteroids, and lower quality of life than other phenotypes. Currently, therapeutic strategies centred on prevention are suggested and the development of resources to assist families with weight loss strategies seems useful for effective weight control and optimal asthma management. Studies on vitamin D supplementation and further knowledge are needed to better define the best therapeutic options to manage asthma in children with overweight/obesity and to reduce the onset and severity of this chronic respiratory disease through the design of a multifactorial intervention.

## 1. Introduction

Obesity and asthma are two of the most significant paediatric health problems worldwide, particularly in industrialized nations, and in recent decades their prevalence has increased dramatically [1,2,3]. Since 2015, the Centers for Disease Control and Prevention (CDC) has listed obesity as a major risk factor for asthma in children [4,5]. The mechanisms that lead to these disorders may start at a paediatric age and include changes in lung mechanics, comorbidities, dietary intake and low physical activity, alterations in insulin and/or glucose metabolism, and systemic inflammation. Moreover, among children with asthma, overweight is associated with a decreased response to drugs and poorer quality of life [6]. This narrative review has the aim to evaluate possible therapeutic strategies to manage asthma in children with overweight/obesity.

## 2. Methods

PubMed was used to search for all of the studies published from January 2008 to June 2018 using the following key words: “asthma” and “overweight” or “obesity” or “obese” and “children” or “paediatric”. As experts in the field, we considered the last ten years as an appropriate time-span for the aim of this narrative review because the problem of asthma in children with overweight/obesity has been particularly considered in this time-frame. More than 100 articles were found, but only those published in English and providing evidence-based data on nutritional approach were included in the evaluation.

## 3. Epidemiology of Obesity and Asthma

In the past four decades, mean BMI and obesity in the paediatric population have significantly increased worldwide. Whilst the trend in children’s and adolescents’ mean BMI has plateaued, albeit at high levels, in many high-income countries since 2000, an increase in mean BMI has been observed in low-income countries [7]. In particular, the global age-standardised prevalence of obesity in childhood and adolescence increased from 0.7% in 1975 to 5.6% in 2016 in girls and from 0.9% in 1975 to 7.8% in 2016 in boys [7].

About 334,000,000 people worldwide have asthma, and it is the most common chronic disease in paediatric age [8]. Asthma is among the top 20 chronic diseases in disability-adjusted life years at paediatric age; at midchildhood ages (5–14 years), it is among the top 10 causes [8]. Asthma has become more common in childhood from the mid-1990s to the mid-2000s. A time trends analysis from the International Study of Asthma and Allergies in Childhood (ISAAC) Phase Three showed increases in global prevalence from 11.1% to 11.6% in children aged 6–7 years and from 13.2% to 13.7% in children aged 13–14 years [9]. There were variations around the world. While asthma has become more common in high-income countries, in many cases, its prevalence was the same or even decreased [9].

Overall, epidemiological data have highlighted the link between obesity and asthma [5,10]. Obesity is a risk factor for incident asthma and it may impact on asthma management [11,12,13]. One meta-analysis of prospective epidemiologic studies in children discovered that asthma incidence increases by 20% in children with overweight and that there is a two-fold higher risk in children with obesity with a dose–response relationship between body weight and incident asthma [14].

Even though overweight/obesity and asthma may simply co-exist in some children, growing evidence underlines the existence of an “obese asthma” phenotype in which high body weight affects and modifies asthma characteristics [15,16]. The “obese asthma” phenotype is complex and multifactorial (Table 1). Nevertheless, several observational analyses have tried to describe its particular molecular and developmental mechanisms [17,18,19,20,21]. Some studies have defined the “obese asthma” phenotype as characterised by additional symptoms, worse control, more frequent and severe acute episodes, reduced response to inhaled corticosteroids, and lower quality of life than other phenotypes [22,23,24,25,26]. Diaz and colleagues described two different “obese asthma” phenotypes categorised by age of onset of asthma symptoms and Th2 inflammation: early-onset and late-onset obese asthma [27].

Children with the early-onset phenotype present asthma symptoms at an age <12 years with an important atopic component, which is characterised by eosinophilic infiltration of the airway and subsequent high exhaled nitric oxide levels [28]. Increased IgE levels, decreased airway function, airway hyperresponsiveness, and poor asthma control also characterize this phenotype. In the early-onset group, obesity may play a complicating role; male and female patients are affected equally. In contrast to early-onset obese asthma, Diaz and colleagues defined patients with late-onset asthma, with characteristics such as females presenting at age >12 years and lacking atopic characteristics. Pulmonary function testing demonstrates that late-onset asthmatic patients have minimal airway obstruction and less airway hyperresponsiveness with better asthma control and lower symptom scores than early-onset asthmatic patients [27]. This late-onset phenotype has a Th2 low profile with predominant neutrophil infiltration as well as low IgE and low eosinophilic infiltration [29]. The authors underlined that both obese asthma phenotypes are characterised by a severe asthma with more exacerbations and poorer symptom control than lean asthmatic patients [27]. 

These findings were confirmed by other analyses [5]. In particular, through a nationwide study of school children, Chen and colleagues examined the influence of long-term adiposity status/short-term adiposity changes on asthma with high or low eosinophilic inflammation after measuring exhaled nitric oxide (FeNO) and atopy, airway inflammation, and lung function. They enrolled 2450 fourth-grade children; new-onset asthma was stratified by airway inflammation status using FeNO. The authors found that long-term adiposity status increased the risk of asthma among children with low FeNO, whereas short-term adiposity changes predicted risks of asthma with high FeNO. Moreover, this cohort study showed that long-term adiposity status reduced pulmonary function, whereas rapid adiposity growth was linked with airway inflammation and atopic diseases [30]. 

However, the mechanisms involved in both obese asthma phenotypes need to be better defined, as their clinical characteristics may simply overlap. Therefore, several analyses have highlighted the need to consider “obese asthma” characterised by difficult-to-control asthma [5]. In a hospital database record study of 74 children aged between 10 and 17 years with asthma, obese patients with early-onset persistent asthma reported clinically worse symptoms than those reported by lean participants. The authors identified that worse characteristics mainly involved shortness of breath, greater short-acting beta-agonists (SABA) use, and worse quality of life. They also found that gastroesophageal reflux scores were higher in obese children and appeared to mediate overweight-related asthma symptoms. In this study, despite a lower fraction of exhaled nitric oxide (FeNO) and reduced methacholine responsiveness, children with obesity needed more than thrice as frequent rescue therapies than lean patients [31]. In another study based on hospital records performed at an urban tertiary children’s hospital, 333 subjects aged 2 to 18 years admitted with asthma were identified. This finding suggested that overweight status was associated with high risk of emergency department (ED) visits in urban children admitted for asthma; in particular, obese preschool subjects were more likely than lean peers to have subsequent ED visits [25].

In a recent retrospective cohort study, a total of 74,338 patient discharges for acute asthma in children 2–18 years old were identified. The authors found that obesity was significantly associated with the use of mechanical ventilation and prolonged length of hospital stay among hospitalized children with acute asthma exacerbation. Their data showed a gap of incremental hospital charges between obese and lean inpatients with asthma, drawing attention to the importance of the prevention of obesity for both clinicians and healthcare payers [24]. Moreover, some research studies have confirmed that increasing adiposity is linked with a higher risk of new-onset asthma in non-atopic children regardless of age of onset of asthma symptoms [32,33]. Lang and co-workers described an example of an “obese asthma” phenotype as a non-atopic child with early life weight gain and subsequent asthma-like symptoms. The authors supposed that the underlying mechanism may be the impaired lung growth and altered airflow perception [34]. 

In paediatric literature, obesity does not seem to lead to a higher risk of allergic airway inflammation, atopic disease, or greater airway reactivity [35,36,37,38,39]. It is known that obesity is associated with a reduced response to inhaled corticosteroids, and this response may be caused by the reduced contribution of eosinophilic disease and/or true glucocorticosteroid resistance [34]. 

Unlike adults, obesity in children and adolescents is not often associated with reduced vital capacity or total lung capacity [17], which may be related to a shorter duration both of obesity and persistent inflammation in the lower airways compared with adults [34]. In some cases, obesity has been linked with greater lung volumes and mild obstructive impairment in airway flows [40,41,42]. Nevertheless, several studies have reported that children with asthma may be at higher risk of obesity [30]. A bidirectional association between asthma and overweight is possible, since many patients with asthma avoid physical activity, increase sedentary time, and swallow oral corticosteroid medications, which are three conditions that facilitate weight gain [2,43,44].

Obesity and asthma also share common comorbidities that complicate and perpetuate the interactions between them. Both asthma and obesity may influence the onset of gastro-oesophageal reflux and sleep disturbances [45,46]. As recently reviewed, such symptoms can mimic the “obese asthma” phenotype and lead to misdiagnosis [5]. Orthopaedic problems are often underrecognized in asthmatic children with obesity. The role of anthropometric indicators and gender in obesity in children with asthma are presented in detail below.

## 4. Anthropometric Indicators of Central Obesity in Asthma

Several studies showed that the distribution of body fat might influence the link between obesity and asthma; in particular, central obesity might determine asthma severity. A cross-observational study was performed with 1362 adolescents 10 to 19 years old of both genders to determine the asthma risk associated with anthropometric indicators of overweight and body fat distribution. The authors found that young participants with asthma had higher rates of excess adiposity according to some indicators of obesity, such as Body Mass Index (BMI-Z) and waist circumference (WC), in females and others, and waist-to-height ratio (WHtR) and the conicity index (CI), in males [47]. It is known that abdominal adiposity may mechanically affect the diaphragm and chest wall compliance with decreased lung volume. Moreover, the accumulation of visceral fat can lead to hyperresponsiveness of the airway through the production of pro-inflammatory markers (e.g., leptin, IL6, and TNF-alpha) and a reduced level of anti-inflammatory markers (e.g., adiponectin) [48]. In non-asthmatic patients, WHtR is linked to the homeostasis model assessment of insulin resistance (HOMA-IR) and is associated with increased lipolysis and atherogenic dyslipidaemia: this result promotes inflammation and the hepatic synthesis of C-reactive protein (CRP) [49,50].

### 4.1. The Influence of Gender

In a recent retrospective analysis, the authors explored the impact of gender on the association between body weight and some asthma phenotypes (allergic, non-allergic, and exercise-induced asthma) [51]. They explored the association between changes in BMI status between birth and the age of 9–11 years and the risk of different asthma phenotypes in 7781 children. They found that allergic asthma was linked to overweight in girls at birth and at 9–11 years, which was in agreement with other studies [52,53]. Changstang and colleagues also found that boys who became obese at the age of 9–11 years had a two-fold greater risk of allergic asthma; in female patients, this association was true for only those children with persistently high body weight. No significant associations were found between BMI changes and non-allergic and exercise-induced asthma [51]. 

It is suggested that the different gender-related associations between obesity and asthma seems to be linked to hormonal characteristics in the prepubertal period (9–11 years), such as those associated with pro-inflammatory hormonal factors (e.g., leptin and adiponectin) and oestrogen [54]. Nevertheless, other studies have described the differing effects of asthma by sex, but their conclusions have been conflicting; thus, further research is required on this topic [55,56,57,58].

### 4.2. The Importance of Diagnosis 

Several studies have provided important findings suggesting the relevant impact of weight reduction in children with obesity and asthma through intensive lifestyle modifications (dietary, physical activities, and behavioural) for the patient and the entire family [5,59]. It is important for health providers to recognize overweight/obese asthmatic children because weight reduction represents a therapeutic option. A recent retrospective cross-sectional design investigated the current practice of recognition, diagnosis, and treatment of high body weight children hospitalized for asthma [60]. The authors evaluated a total of 510 patients aged 3 to 17 years admitted to the hospital with asthma and calculated BMI and BMI percentile through an analysis of patient height, weight, age, and gender; they found that 19.6% of patients were obese and that 13.3% of patients were overweight. Nevertheless, a review of the records showed that data of BMI percentile by the provider were missing in 493 (96.7%) of the records in the total sample; in particular, BMI percentile was recorded in only 3.3% of all charts, in only 11% of children with obesity, and in 0% of subjects with overweight. BMI percentile was documented more often in patients with severe obesity and moderate to severe asthma. The results also suggested that the relationship between asthma severity and overweight was more evident in older subjects, supporting the importance of recognizing high body weight in young children. In this study, only 8% of asthmatic children with obesity were treated. The authors underlined that overweight and obesity were underrecognized, underdiagnosed, and undertreated in patients hospitalized for asthma [60].

## 5. Therapeutic Options

Table 2 summarizes asthma management in children with obesity. At present, the management of asthma in children with obesity, in a step-up strategy, typically requires the use of leukotriene receptor inhibitors and inhaled corticosteroids with long-acting b2-agonsits. However, treatment plans need to be multidisciplinary, including not only asthma medications but also weight management, dietary changes, and mental health care.

### 5.1. Asthma Management: Different Responsiveness to Asthma Therapy in Children with Obesity

According to guidelines from Europe and the USA, asthma is acutely treated with a short-acting b2-agonist, such as albuterol, which induces bronchodilation and provides acute relief of asthma symptoms by relaxing smooth muscles in the airways. Depending on the severity of asthma symptoms and exacerbations, asthma is controlled in a step-up or step-down strategy with daily drugs, such as leukotriene receptor inhibitors, inhaled corticosteroids, and a combination of inhaled corticosteroids with long-acting b2–agonists [61]. 

McGarry and colleagues performed a cross-sectional study on the association of bronchodilator responsiveness in obese compared with non-obese children and adolescents with asthma [62]. The subjects were enrolled from the Study of African Americans, Asthma, Genes, and Environments (SAGE II) and the Genes-environments and Admixture in Latino Americans (GALA II) study, which are two identical, parallel, case-control studies of asthma in black and Latino Americans, respectively. The authors enrolled 2963 patients with asthma between 8 and 21 years of age who underwent spirometry from 2008 to 2013. In their analysis, 36% of the subjects were obese, with a slightly lower prevalence of obesity in girls than in boys (35% vs. 36.8%, respectively). They found that obesity was not associated with bronchodilator response; in particular, the odds of being bronchodilator unresponsive were 24% greater among obese children and adolescents than among their lean counterparts. In addition, McGarry and colleagues analysed how asthma controller medication use differed between obese and non-obese subjects when stratified by bronchodilator responsiveness. They discovered that obese subjects were 33% more likely to be prescribed leukotriene receptor inhibitors than non-obese subjects in the bronchodilator unresponsive group. Obese subjects were 37% more likely to be on an inhaled corticosteroid with a long-acting b2-agonist than lean patients in the bronchodilator unresponsive group. In conclusion, the authors found that after adjusting for other factors that influence bronchodilator response, obesity was associated with bronchodilator unresponsiveness. They also defined that among the bronchodilator unresponsive group, obese children and adolescents presented worse asthma control and increased asthma morbidity than lean subjects. In particular, they suffered more from wheezing, were awakened at night from their asthma symptoms, and more likely needed leukotriene receptor inhibitors and inhaled corticosteroids with long-acting b2–agonists [62].

Several studies also analysed the influence of BMI on the response to asthma controller medications. In particular, Peters-Golden and colleagues performed a post hoc analysis, pooling data from four double-blind, placebo-controlled studies randomising 3073 moderate asthmatic patients to montelukast, beclomethasone, or placebo [63]. Evaluations were conducted using BMI classification into normal, overweight, and obese categories with BMI as a continuous variable. Males and females aged over 15 years old with a more than one year history of clinical symptoms of asthma were enrolled. The aim of their study was to provide evidence for the differential efficacy of montelukast and inhaled beclomethasone in subjects with differing BMI. The primary end point was asthma control days; other end points were forced expiratory volume in one second, b-agonist use, and nocturnal awakening. The treatment groups were balanced for BMI, demographic characteristics, and parameters of asthma control. In this study, an analysis of the placebo-treated patients showed that high body weight adversely influenced the natural history of asthma during the 6 or 12 weeks of follow-up care. In addition, an evaluation of montelukast- and beclomethasone-treated subjects suggested that BMI could influence responsiveness to asthma therapy. The effect of montelukast on an asthma control day, defined as a day with no more than two puffs of b-agonist use, no night-time awakenings, and no asthma exacerbations, was similar across all three BMI categories, but the effect of beclomethasone decreased with increasing body weight [63]. Therefore, montelukast should be considered as an add-on therapeutic strategy if asthma control is not achieved with corticosteroid inhalator therapy alone [34].

In another analysis, Chen and colleagues also investigated the effects of asthma and related phenotypes on the development of obesity in a cohort of non-obese children (ages 5, 2–7, and 9 years) participating in the southern California Children’s Health Study (CHS). The authors examined obesity incidence over a 10-year follow-up and performed a replication analysis in an independent cohort of CHS children who were lean at the beginning and were followed from mean age of 9, 7 to 17, and 8 years. The authors found that during the follow-up, 342 (15.8%) children developed obesity. In particular, non-obese children with asthma at baseline were 51% more likely to develop obesity during follow-up compared with children without asthma at baseline, and children with a history of wheeze before study entry were also at a 42% higher risk of becoming overweight during follow-up compared to children who never experienced wheeze. Moreover, patients who used asthma rescue drugs at study entry had a significantly lower risk of becoming obese during the follow-up compared with participants who did not use asthma therapies. The authors underlined that the use of controller and steroid medications was not associated with the risk of developing obesity independently from physical activity and other asthma medication use. Their results suggested that using asthma rescue medication in early childhood might protect against weight gain in later life. Thus, early diagnosis and treatment of asthma may avoid the vicious cycle of asthma increasing the development of obesity with high body weight, subsequently inducing worse asthma symptoms and morbidity, which leads to further weight gain [30]. In conclusion, Chen and colleagues identified in asthma therapies the potential to prevent obesity through the early diagnosis and treatment of childhood asthma [30]. Other authors wrote that long-term treatment with glucocorticosteroids in asthmatic subjects could influence lipid metabolism by increasing the uptake of lipids from the digestive system and enhancing lipid storage in tissues [64], but their results have not been consistent [65].

### 5.2. Weight Loss in Obese Asthmatic Children

As obesity influences several asthma parameters, the consequence of weight reduction in overweight/obese asthmatic children could be an important target for current treatment approaches and has been investigated in several studies [66,67]. Some authors defined obesity as one of the few modifiable risk factors for asthma, highlighting the importance of incorporating a weight reduction program into the guidelines for childhood asthma [67]. In fact, weight reduction in asthmatic children might lead to an improved asthma prognosis via improved asthma-related quality of life, asthma control, and lung function. In a two-armed randomised controlled trial, the Mikado study, 87 children were recruited via online questionnaires, pulmonary paediatricians, the youth department of the municipal health services, and cohorts of existing studies. All participants were 6–16 years old with current asthma and a BMI in the overweight or obesity range without comorbidities (such as diabetes or heart diseases). Children in the intervention arm received a multifactorial intervention lasting for 18 months, consisting of sessions concerning sports, parental involvement, and individual counselling and lifestyle advice, including dietary advice and cognitive behavioural therapy. The control group received usual care. Regular measurements were performed every six months. The primary outcome variables were forced expiratory volume 1 s (FEV1) and body mass index-standard deviation score (BMI-SDS). Secondary outcomes included other lung function parameters, asthma control, asthma-specific quality of life, use of asthma medication, and markers of systemic inflammation and airway inflammation. This study showed that overweight/obese subjects with asthma decreased in body weight and BMI-SDS, with clinically important improvements in all lung function indices, asthma control, aerobic fitness, and asthma quality of life (PAQLQ), although improvements were no greater in the intervention than in the control group. Only forced vital capacity (FVC%) improved significantly more in the intervention group than in the control group, and significant improvements in asthma control and asthma-related quality of life were more evident in the intervention group than in the control group. The authors explained the lack of contrast between the intervention and control group through the motivation to lose weight in the control group in which participants sought out extraprofessional help from a dietician, a weight reduction consultant, or online weight reduction advice during the study. This study also underlined that deteriorations in body weight/BMI-SDS and lung function in children with overweight/obesity and asthma can be reduced or even reversed [67]. 

In the study by Da Silva and colleagues, 26 children with high body weight and asthma aged 15–19 years old were enrolled in either a year-long psychological, nutrition, exercise, and medical intervention or educational control [68,69]. The subjects underwent psychological and nutritional counselling once a week and exercised three times weekly. Lung function, anthropometrics, maximal oxygen uptake (VO_2_max), asthma severity, fat percentage, and systemic markers (leptin, adiponectin, and CRP) were measured at baseline, 6 months, and 12 months. The intervention group had significantly decreased body fat percent, visceral fat, weight, and BMI compared with the control group and displayed reduced leptin and CRP and improved asthma control and lung function. Increased values of adiponectin in the intervention group were associated with improved lung function [68].

### 5.3. The Role of Physical Activity

The WHO and many nations have established that children should spend a minimum of 60 min daily in at least moderately intense physical activity because it plays a critical role in adequate growth and development, is a useful therapy for chronic diseases, and reduces several adult bone and cardiometabolic diseases with paediatric origins [70]. In a systematic review, Leinaar and colleagues analysed the relationship between asthma and overweight or obesity in youth, considering the role of physical activity as a mediator in this relationship. They found that physical activity might be deterred by symptoms of asthma, predisposing asthmatic children to decreased physical exercise and subsequent weight gain. Several possible determinants could be implicated in the association between asthma and physical activity in children, such as symptoms of asthma or exercise-induced asthma, negative self-perception of physical ability, and parental perceptions of risk associated with physical exercise in asthmatic youth. They were identified as possible factors disinclining children to pursue physical activity [71]. 

Moreover, Chen and colleagues suggested that physical activity should be encouraged to promote pulmonary function, especially in long-term obese children, who may present asthma through a decline in airway function. For those patients with a history of atopic disease or airway inflammation, the main strategies should be centred on the prevention of rapid weight gain during adolescence to avoid asthma development [72]. 

In a recent analysis, baseline data from 324 urban children with poorly controlled asthma (3–10 years) enrolled in the School-Based Telemedicine Enhanced Asthma Management program in Rochester, New York, were analysed. Few children (39%) participated in 1 or more hour of physical exercise per day. In addition, most (85%) did not walk to and from school, 38% did not have any recess in school, and 35% reported no safe place to exercise. More children with very poorly controlled asthma symptoms than children with milder symptoms reported limitations in gym class and even in mild activities. Children with activity limitations were at significantly greater risk of being overweight or obese. The authors emphasised that efforts are needed to optimize asthma control and provide opportunity for increased physical activity inside and outside of school [73].

In a cross-sectional study, school-aged children (*n* = 122) were divided in four groups (healthy control, asthma, overweight/obesity and asthma, overweight/obesity); they were asked to perform lung functions tests and wear an activity monitor for 7 days. The authors did not find significant associations between asthma, overweight, and physical exercise levels in school-aged children compared with peers without asthma and/or overweight/obese. Although physical activity did not seem to be correlated to current asthma, the authors emphasised that physical exercise levels were alarmingly low in the entire study population, leading to higher health risks. They also emphasized the need to further investigate the role of modifiable risk factors, such as physical activity and diet, in the management of children with the difficult-to-treat “obese asthma” phenotype [74].

### 5.4. Diet-Induced Weight Loss

In asthmatic patients with obesity, the specific content of the diet, including fats, sugar, and low nutrients, may contribute to the chronic inflammatory state. Several studies have investigated the role of dietary supplementations or alterations as a means to develop better asthma control [27]. 

In a recent 10-week pilot randomized controlled trial, obese asthmatic children aged 8–17 years were randomized to a wait-list control or diet-induced weight loss group. Lung function, Asthma Control Questionnaire score, sputum, and systemic inflammation were assessed at baseline and postintervention. Jensen and colleagues found that dietary intervention induced acute weight loss with subsequent improvements in static lung function and asthma control, whereas systemic and airway inflammation did not change following weight loss. However, changes in BMI z-score were associated with changes in airway and systemic inflammation [75]. The authors underlined the role of diet-induced weight loss to achieve significant improvements in clinical outcomes for obese children with asthma [75].

The evaluation of dietary components may constitute an additional therapeutic option for obese asthmatic children. Several studies have shown that some dietary characteristics, such as fish, omega-3 fatty acids, fresh fruits, vegetables, and low saturated fat content, might contribute to reduced risk of asthma and improved control of existing asthma [76]. In particular, it is known that intake of omega-3 fatty acids may improve asthma control in obese asthmatic patients through several anti-inflammatory mechanisms; therefore, some authors evaluated its use specifically in obese asthmatic patients with persistent disease [77]. In addition, several analyses found that pretreatment with omega-3 fatty acid supplements prior to physical activity might lead to reduced asthma symptoms and exercise-related drop in lung function [78,79].

### 5.5. The Potential Role of Vitamin D

In a recent review of the literature, the potential role of vitamin D status in the link between obesity and asthma severity/control in children was analysed [80]. The authors found that obese children and adolescents with asthma had low vitamin D status with a higher risk of acute respiratory infections and decreased corticosteroid responsiveness. They also underlined that vitamin D deficiency increased as weight increased [80]. 

There is growing evidence to support that vitamin D supplementation can improve asthma severity and control. A meta-analysis of five clinical trials among children with asthma suggested a statistically significant reduction in asthma exacerbation with vitamin D therapy. However, the dose range of vitamin D varied considerably from 500 to 2000 UI per day [81]. In a recent randomized trial comparing vitamin D3 supplements (800 IU/day) with placebo for 2 months, 89 schoolchildren with asthma were randomly assigned to receive vitamin D (*n* = 54) or placebo (*n* = 35). After 2 months, asthma control was significantly more improved in the vitamin D group compared with the placebo group. The authors suggested that daily low-dose, short-term vitamin D supplementation in addition to standard treatment might improve levels of asthma control in schoolchildren [82]. Thus far, data from randomized trials are limited; therefore, there is not enough evidence to recommend vitamin D therapy for improving asthma management. Additionally, future research should investigate the role of vitamin D supplementation for improving asthma severity/control among obese children with asthma and low vitamin D status.

### 5.6. Pharmacotherapy

As recently reviewed, no specific medications are recommended for obesity in paediatric patients with asthma, as children are treated according to the frequency and severity of symptoms and their impact on daily activities. Weight loss drugs are recommended only in children with severe obesity-related complications [34,83]. Orlistat is currently the only widely available and approved medication for simple weight loss in children, and little is known about its effect on asthma. It impairs triglyceride hydrolysis and can block up to 30% of fat absorption in the diet, reducing serum low-density lipoprotein and total cholesterol and improving glycaemic control. Gastrointestinal side effects can limit adherence and the drug’s long-term effectiveness, and safety data beyond two years are limited [34]. Metformin is another medication that needs further investigation in the setting of obese asthma. Its use is approved for obese adolescents with type 2 diabetes, but it is not specifically approved for use in children for simple weight loss. Metformin typically induces significant weight loss by means of increasing satiety and decreasing intestinal nutrient absorption. Nevertheless, studies evaluating metformin in paediatric patients have been few in number, small in size, and short in duration [34].

## 6. Conclusions

Both overweight/obesity and asthma are significant paediatric health problems worldwide, and their increasing incidence has led healthcare providers to carefully evaluate their association. Obesity and asthma are complex disorders characterised by a chronic inflammatory state that can affect quality of life. In particular, it is known that high body weight is a risk factor for incident asthma and complicates its diagnosis and management. Obesity and asthma share genetic, developmental, lung mechanical, immunological, and behavioural factors that can clarify their association. However, their complex mechanistic interactions are still poorly understood, and the temporal associations between them remains unclear. Several studies have shown that overweight/obesity often precedes asthma symptoms and that comorbidities due to high body weight may worsen or even mimic asthma characteristics, leading to misdiagnosis and mistakes in the design of therapeutic strategies. Therefore, it is important to be careful in using the correct data to make an early diagnosis, such as the evaluation of BMI to define obesity [83] and evidence of excessive variability in expiratory lung function, in subjects with typical respiratory symptoms [61].

Several authors have described an “obese asthma” phenotype characterised by additional symptoms, worse control, more frequent and severe exacerbations, reduced response to inhaled corticosteroids, and lower quality of life. This phenotype can also be divided into early- and late-onset obese asthma, categorised by the age of onset of asthma symptoms and Th2 inflammation. In particular, the late-onset obese asthma phenotype seems to be associated with non-Th2 signalling pathways. A better understanding of the mechanisms mediating these obese asthma phenotypes would have significant implications for therapeutic strategies.

Overall, in children with obesity and asthma, therapeutic strategies centred on prevention are suggested and it is useful to develop resources to assist families in weight loss strategies that include appropriate lifestyle changes (increasing exercise and improving adherence to dietary guidelines) for effective weight control and optimal asthma management. Although this recommendation can be generalized to the whole population of paediatric patients with asthma and overweight/obesity, primary care paediatricians and general practitioners should consider that specific populations (i.e., ethnic minorities) could find difficulties in changing their lifestyle. Future studies should clarify the role of vitamin D supplementation for improving asthma management among children and adolescents with asthma.

## Figures and Tables

**Table 1:** “Obese asthma” phenotypes.

Obese Asthma Phenotypes	Early-Onset Asthma	Late-Onset Asthma
Age at onset	<12 years	>12 years
Gender	Female = Male	Female > Male
Airway function (FEV1, FVC)	Severe decrease in airway function	Minimal airway obstruction
Nature	Atopic	Non-atopic
Airway hyperreactivity	Severe airway Hyperresponsiveness	Less airway Hyperresponsiveness
Symptom score	High symptom score	Low symptom score
Airway inflammation	Eosinophilic airway infiltration	Neutrophilic infiltration
Th1-Th2 profile	High Th2 biomarkers	Low Th2 biomarkers

**Table 2:** The best therapeutic options in obese children with asthma.

The Best Therapeutic Options
**key points** Recognise and diagnose overweight/obesity in asthmatic children.
Be aware that “obese asthma” phenotypes are characterised by more symptoms, worse control, more frequent and severe exacerbations, reduced response to corticosteroids, and lower quality of life.
**how to treat**
**Management of asthma with early interventions and a step-up strategy:** obese asthmatic children are more often bronchodilator unresponsive; they need controller medications and exhibit a better response to leukotriene receptor inhibitors.
**Weight loss through multidisciplinary intervention:** increasing physical activity, improving adherence to dietary guidelines, evaluating vitamin D supplementation; using specific medications (Orlistat or Metformin) only in children with severe obesity-related complications.
**the best strategy** Prevention of paediatric obesity by promoting healthy diet, activity, and environment.
